# Supplementary material for: Allochthonous Trichoderma Isolates Boost Atractylodes lancea Herb Quality at the Cost of Rhizome Growth
Source: J Fungi (Basel). 2024 May 14;10(5):351. doi: 10.3390/jof10050351 (PMC11122596; doi:10.3390/jof10050351)
Supplement: Supplementary file 1 [file jof-10-00351-s001.zip › Supplementary Figure S2 .docx]

**
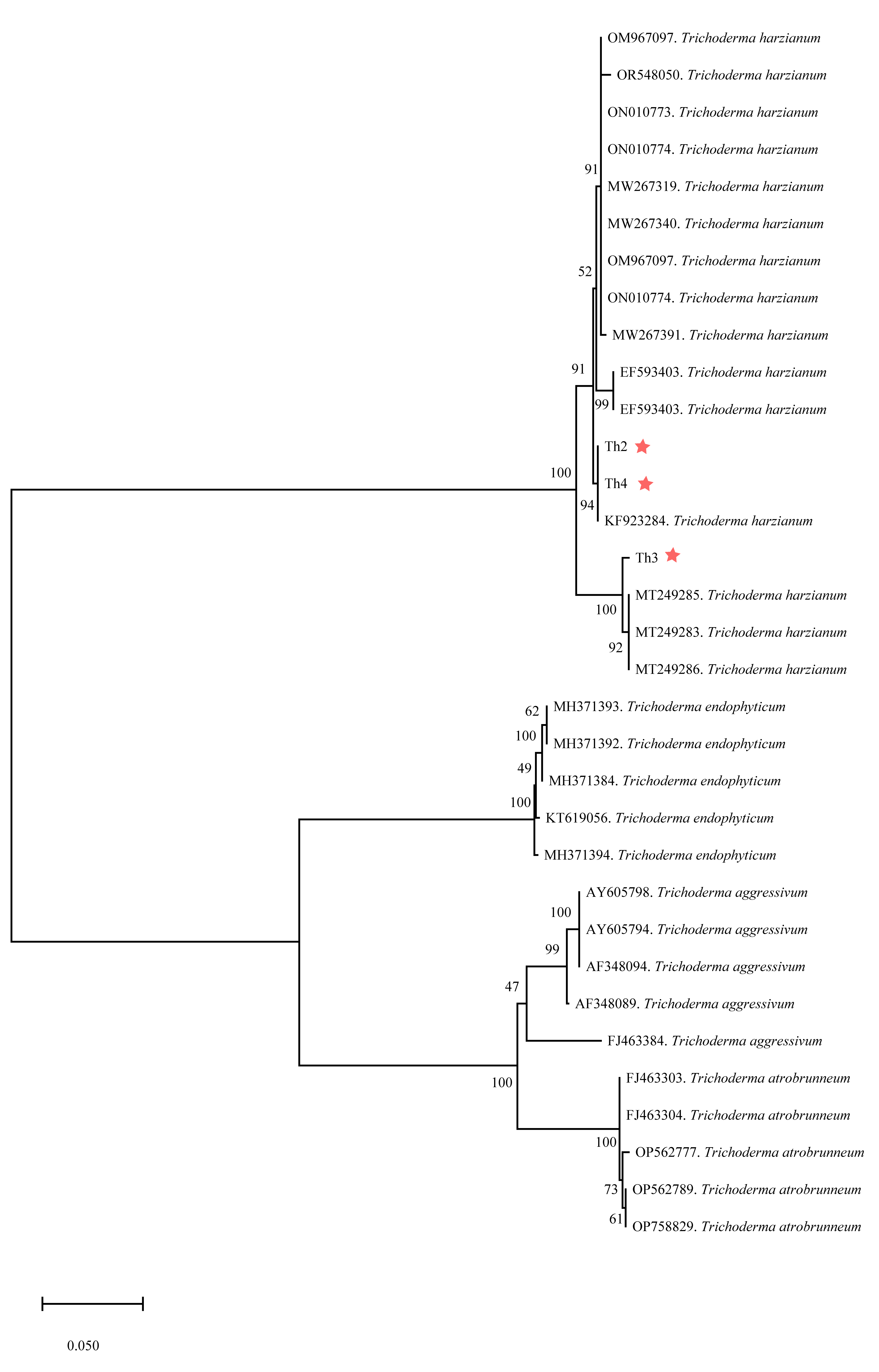
**

**Supplementary Figure S2**. Phylogenetic tree of Th2, Th3, Th4 and related strains constructed based on their TEF1 sequences.
