# Supplementary material for: Allochthonous Trichoderma Isolates Boost Atractylodes lancea Herb Quality at the Cost of Rhizome Growth
Source: J Fungi (Basel). 2024 May 14;10(5):351. doi: 10.3390/jof10050351 (PMC11122596; doi:10.3390/jof10050351)
Supplement: Supplementary file 1 [file jof-10-00351-s001.zip › Supplementary Table S1 Data of figure 5.docx]

Table S1 Data of figure 5

| Treatment | Fold increase of total weight | root-shoot ratios | Rhizome dry weight(g) |
| --- | --- | --- | --- |
| CK | 1.157±0.801c | 2.779±0.818b | 0.1708±0.080a |
| Fo+Fs | 3.253±1.173a | 4.646±0.750a | 0.150±0.053ab |
| Fo+Fs_Th2 | 1.571±0.697bc | 2.647±0.714b | 0.111±0.055ab |
| Th2_Fo+Fs | 2.489±1.162ab | 3.014±0.785b | 0.109±0.039ab |
| Th2 | 1.096±0.458bc | 2.869±0.946b | 0.088±0.041b |

| Treatment | Fold increase of total weight | root-shoot ratios | Rhizome dry weight(g) |
| --- | --- | --- | --- |
| CK | 1.157±0.801b | 2.779±0.818b | 0.1708±0.080b |
| Fo+Fs | 3.253±1.173a | 4.646±0.750ab | 0.150±0.053b |
| Fo+Fs_Th3 | 3.741±0.800a | 4.583±0.498a | 0.236±0.072a |
| Th3_Fo+Fs | 2.991±0.866a | 5.986±1.277a | 0.245±0.097a |
| Th3 | 0.868±0.295b | 1.382±0.465c | 0.078±0.033c |

| Treatment | Fold increase of total weight | root-shoot ratios | Rhizome dry weight(g) |
| --- | --- | --- | --- |
| CK | 1.157±0.801b | 2.779±0.818b | 0.1708±0.080a |
| Fo+Fs | 3.253±1.173a | 4.646±0.750a | 0.150±0.053a |
| Fo+Fs_Th3 | 1.029±0.707b | 1.158±0.561c | 0.034±0.013b |
| Th3_Fo+Fs | 0.893±0.286b | 2.483±1.024b | 0.075±0.033b |
| Th3 | 2.783±2.125a | 2.857±1.076b | 0.144±0.046a |

^1^ Different lowercase letters represent significant differences between the samples of different treatments on the same day. All significances were at *P* < 0.05.
