# Supplementary material for: Allochthonous Trichoderma Isolates Boost Atractylodes lancea Herb Quality at the Cost of Rhizome Growth
Source: J Fungi (Basel). 2024 May 14;10(5):351. doi: 10.3390/jof10050351 (PMC11122596; doi:10.3390/jof10050351)
Supplement: Supplementary file 1 [file jof-10-00351-s001.zip › Supplementary Table S2 Data of figure 6.docx]

Table S2 Data of figure 6 (mg/g DW)

| Treatment | hinesol | β-eudesmol | atractylon | atractylodin |
| --- | --- | --- | --- | --- |
| CK | 0.000992±0.0000858d | 0.00245±0.00114c | 0.232±0.0744b | 0.356±0.111b |
| Fo+Fs | 0.00362±0.000628a | 0.00545±0.00903a | 3.904756±0.931a | 4.72±1.06a |
| Fo+Fs_Th2 | 0.00305±0.00103ab | 0.00487±0.0147ab | 3.26±1.750a | 4.14±2.52a |
| Th2_Fo+Fs | 0.00198±0.000247c | 0.00318±0.00218bc | 1.128±0.227b | 1.44±0.431a |
| Th2 | 0.00220±0.000939bc | 0.00581±0.0266a | 0.586±0.330b | 0.620±0.325b |

| Treatment | hinesol | β-eudesmol | atractylon | atractylodin |
| --- | --- | --- | --- | --- |
| CK | 0.000992±0.0000858b | 0.00245±0.00114b | 0.232±0.0744c | 0.356±0.111b |
| Fo+Fs | 0.00362±0.000628a | 0.00545±0.00903a | 3.904756±0.931a | 4.72±1.06a |
| Fo+Fs_Th3 | 0.00262±0.000665a | 0.00366±0.00639b | 1.67±0.692b | 3.20±1.00a |
| Th3_Fo+Fs | 0.00286±0.000818a | 0.00385±0.00664b | 2.00±0.834b | 3.63±1.38a |
| Th3 | 0.00261±0.00226a | 0.00667±0.0283a | 4.22±2.51a | 3.75±2.62a |

| Treatment | hinesol | β-eudesmol | atractylon | atractylodin |
| --- | --- | --- | --- | --- |
| CK | 0.000992±0.0000858b | 0.00245±0.00114c | 0.232±0.0744c | 0.356±0.111b |
| Fo+Fs | 0.00362±0.000628b | 0.00545±0.00903b | 3.904756±0.931a | 4.72±1.06a |
| Fo+Fs_Th4 | 0.00304±0.000808a | 0.00574±0.0188b | 1.47±1.18bc | 1.15±0.626b |
| Th4_Fo+Fs | 0.00368±0.00101a | 0.00843±0.0211a | 2.91±2.60ab | 3.25±2.78a |
| Th4 | 0.00171±0.000807b | 0.00400±0.0192bc | 0.509±0.418c | 0.763±0.673b |

^1^ Different lowercase letters represent significant differences between the samples of different treatments on the same day. All significances were at *P* < 0.05.
