# Supplementary material for: Allochthonous Trichoderma Isolates Boost Atractylodes lancea Herb Quality at the Cost of Rhizome Growth
Source: J Fungi (Basel). 2024 May 14;10(5):351. doi: 10.3390/jof10050351 (PMC11122596; doi:10.3390/jof10050351)
Supplement: Supplementary file 1 [file jof-10-00351-s001.zip › Supplementary Table S3 Data of figure 8.docx]

Table S3 Data of figure 8

| *Trichoderma*  strain | Culturing  regimes | 2 d colony diameter  (cm) | 10 d conidia concentration  (*10^7^ cfu /mL) |
| --- | --- | --- | --- |
| Th2 | PDA | 7.300±0.110a | 1.317±0.202b |
|  | PSA | 7.250±0.105a | 2.983±0.153ab |
|  | SDA | 6.733±0.121b | 0.683±0.126b |
|  | CZA | 4.100±0.167c | 1.983±2.225ab |
|  | CMA | 6.750±0.055b | 4.267±0.660a |
|  | RBA | 3.633±0.137d | 2.033±2.019ab |
| Th3 | PDA | 7.733±0.216a | 4.833±0.289c |
|  | PSA | 7.717±0.133a | 21.1±7.532b |
|  | SDA | 8.050±0.084a | 8.833±3.014c |
|  | CZA | 3.683±0.319d | 13.817±3.147bc |
|  | CMA | 7.267±0.333b | 37.333±1.443a |
|  | RBA | 4.767±0.052c | 22.167±10.484b |
| Th4 | PDA | 7.733±0.163a | 0.567±0.225d |
|  | PSA | 7.233±0.121b | 2.033±0.076b |
|  | SDA | 6.783±0.117c | 0.717±0.225d |
|  | CZA | 3.067±0.137e | 1.35±0.0500c |
|  | CMA | 6.683±0.098c | 13.500±0.500a |
|  | RBA | 3.433±0.197d | 0.567±0.076d |
| Th2 | L | 5.796±0.546b | 0.717±0.275b |
|  | D | 7.300±0.100a | 1.317±0.202a |
|  | L/D | 5.968±0.452b | 0.600±0.132b |
| Th3 | L | 7.671±0.314a | 0.277±0.146c |
|  | D | 7.733±0.197a | 4.833±0.289a |
|  | L/D | 7.682±0.506a | 1.100±0.304b |
| Th4 | L | 6.836±0.287b | 0.567±0.225a |
|  | D | 7.733±0.149a | 0.567±0.225a |
|  | L/D | 7.136±0.252b | 0.667±0.247a |
| Th2 | 15 ℃ | 1.249±0.126d | 0.550±0.150b |
|  | 20 ℃ | 5.013±0.181b | 0.367±0.076b |
|  | 25 ℃ | 7.300±0.0500a | 1.317±0.202a |
|  | 30 ℃ | 7.400±0.0570a | 0.550±0.087b |
|  | 35 ℃ | 3.060±0.416c | 0.450±0.132b |
|  | 40 ℃ | 0 | 0 |
| Th3 | 15 ℃ | 1.061±0.265d | 0.983±0.144bc |
|  | 20 ℃ | 5.503±0.124c | 4.333±1.756bc |
|  | 25 ℃ | 7.733±0.236b | 4.833±0.289b |
|  | 30 ℃ | 9.000±0.00a | 9.000±5.408a |
|  | 35 ℃ | 5.477±0.595c | 0.500±0.087bc |
|  | 40 ℃ | 0 | 0 |
| Th4 | 15 ℃ | 0.805±0.084c | 0.417±0.126a |
|  | 20 ℃ | 4.012±0.208b | 0.467±0.161a |
|  | 25 ℃ | 7.733±0.153a | 0.567±0.225a |
|  | 30 ℃ | 7.398±0.437a | 0.583±0.189a |
|  | 35 ℃ | 7.262±0.474a | 0.550±0.100a |
|  | 40 ℃ | 0 | 0 |
| Th2 | 5 | 5.832±0.149b | 0.817±0.176a |
|  | 6 | 6.905±0.283a | 1.033±0.076a |
|  | 7 | 6.163±0.278b | 0.950±0.173a |
|  | 8 | 4.630±0.355cd | 0.367±0.029b |
|  | 9 | 4.752±0.084c | 0.317±0.029b |
|  | 10 | 4.305±0.170d | 0.467±0.161b |
| Th3 | 5 | 7.373±0.316ab | 16.667±6.714b |
|  | 6 | 7.787±0.080a | 25.667±2.930a |
|  | 7 | 7.017±0.410b | 30.167±5.795a |
|  | 8 | 4.837±0.327cd | 2.433±0.202d |
|  | 9 | 5.267±0.430c | 10.000±2.291bc |
|  | 10 | 4.298±0.377d | 7.000±0.500cd |
| Th4 | 5 | 7.693±0.263b | 0.717±0.076e |
|  | 6 | 8.757±0.421a | 1.050±0.150e |
|  | 7 | 7.275±0.604b | 23.000±1.000a |
|  | 8 | 7.275±0.161b | 3.700±0.312d |
|  | 9 | 4.658±0.400d | 11.167±1.155b |
|  | 10 | 5.590±0.100c | 6.667±0.289c |
| Th2 | sucrose | 4.100±0.173d | 1.983±2.225c |
|  | glucose | 6.200±0.051a | 10.667±0.764a |
|  | D-fructose | 4.780±0.053b | 5.167±1.429b |
|  | soluble starch | 6.058±0.053a | 9.833±0.577a |
|  | maltose | 6.032±0.053a | 7.000±0.500b |
|  | D-mannose | 4.620±0.053b | 10.500±0.866a |
|  | No carbon sourse | 4.305±0.203c | 5.500±0.500b |
| Th3 | sucrose | 3.683±0.176c | 13.817±3.147ab |
|  | glucose | 6.212±0.171a | 8.833±0.764de |
|  | D-fructose | 5.053±0.067b | 14.833±1.528a |
|  | soluble starch | 6.147±0.137a | 6.333±0.289d |
|  | maltose | 5.822±0.215a | 11.833±0.764bc |
|  | D-mannose | 4.718±0.277b | 10.500±1.000cd |
|  | No carbon sourse | 3.895±0.335c | 6.333±0.289d |
| Th4 | sucrose | 3.067±0.104f | 1.350±0.050e |
|  | glucose | 6.262±0.186b | 9.167±0.764b |
|  | D-fructose | 5.093±0.044c | 3.983±0.448d |
|  | soluble starch | 6.998±0.134a | 8.000±1.323bc |
|  | maltose | 6.250±0.046b | 12.333±2.021a |
|  | D-mannose | 4.815±0.017d | 12.667±2.93a |
|  | No carbon sourse | 4.082±0.083e | 5.500±0.500cd |
| Th2 | beef extract | 6.487±0.028b | 3.333±0.577c |
|  | peptone | 6.132±0.305b | 4.167±0.289c |
|  | ammonium dihydrogen phosphate | 7.507±0.18a | 12.667±2.517a |
|  | yeast extract | 5.602±0.076c | 3.500±0.500a |
|  | ammonium sulfate | 6.393±0.240b | 7.667±0.764b |
|  | ammonium nitrate | 4.100±0.173d | 1.983±2.225c |
|  | No nitrogen source | 4.428±0.214d | 1.833±0.289c |
| Th3 | beef extract | 6.802±0.038b | 9.000±2.000b |
|  | peptone | 6.778±0.044b | 8.833±0.764b |
|  | ammonium dihydrogen phosphate | 7.392±0.053a | 10.667±0.289b |
|  | yeast extract | 6.277±0.400c | 8.000±1.000b |
|  | ammonium sulfate | 6.752±0.220b | 4.667±0.764c |
|  | ammonium nitrate | 3.683±0.176e | 13.817±3.147a |
|  | No nitrogen source | 4.555±0.061d | 3.833±0.764c |
| Th4 | beef extract | 6.883±0.077a | 8.833±1.528bc |
|  | peptone | 5.923±0.05c | 4.333±0.289d |
|  | ammonium dihydrogen phosphate | 7.072±0.045a | 10.667±0.289b |
|  | yeast extract | 6.165±0.095b | 13.000±2.291a |
|  | ammonium sulfate | 5.778±0.208c | 7.667±0.289c |
|  | ammonium nitrate | 3.067±0.104e | 1.350±0.050e |
|  | No nitrogen source | 4.22±0.106d | 2.667±0.289de |

^1^ Different lowercase letters represent significant differences between the samples of different treatments on the same day. All significances were at *P* < 0.05.
