# Supplementary figures and images for: Allochthonous Trichoderma Isolates Boost Atractylodes lancea Herb Quality at the Cost of Rhizome Growth
Source: J Fungi (Basel). 2024 May 14;10(5):351. doi: 10.3390/jof10050351 (PMC11122596; doi:10.3390/jof10050351)

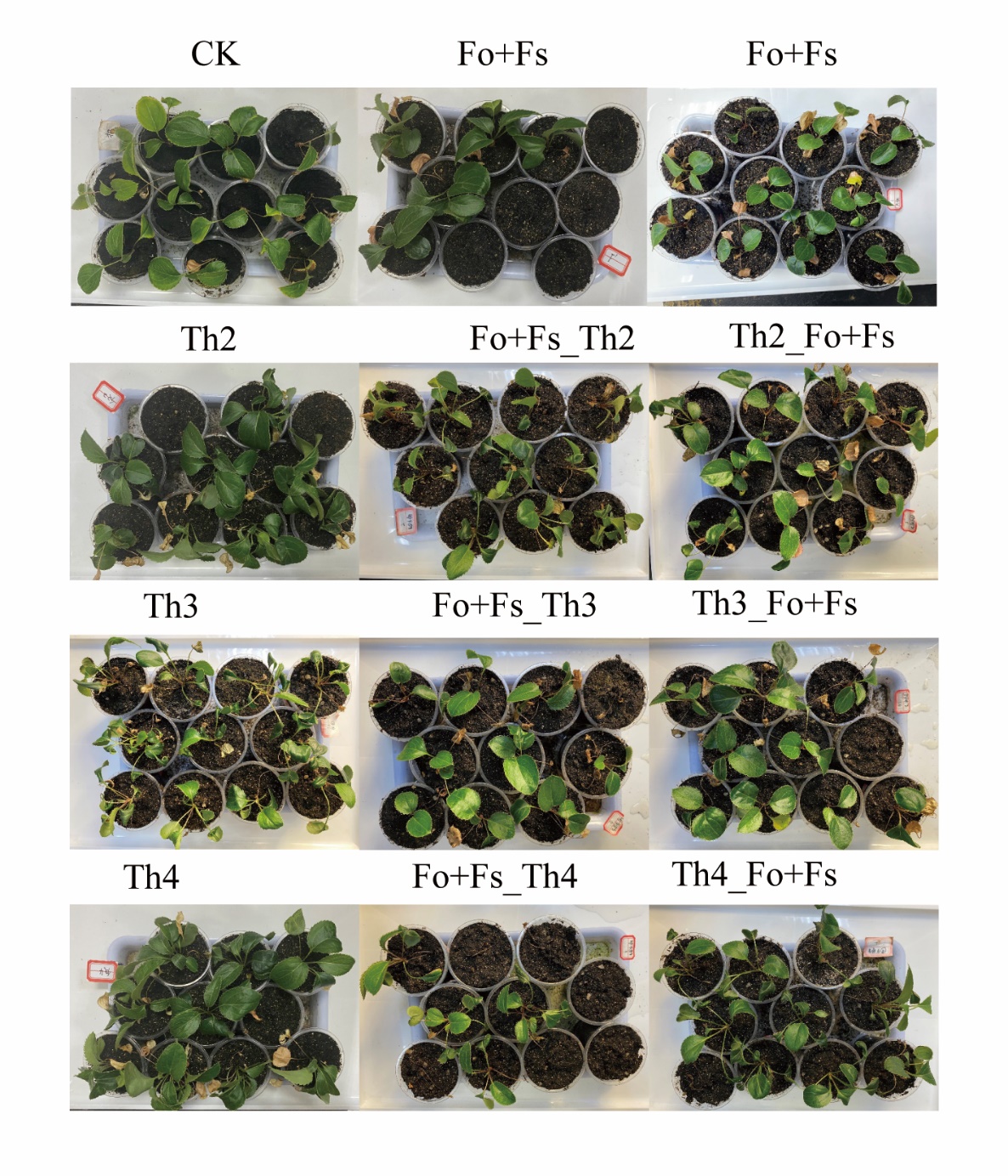
 **Supplementary Figure S1.** Photographs of 70-day-old seedlings right before harvesting.

Supplement: Supplementary file 1 [file jof-10-00351-s001.zip › Supplementary Figure S1.docx]
